# On the combination of two visual cognition systems using combinatorial fusion

**DOI:** 10.1007/s40708-015-0008-0

**Published:** 2015-02-03

**Authors:** Amy Batallones, Kilby Sanchez, Brian Mott, Cameron Coffran, D. Frank Hsu

**Affiliations:** 1 Laboratory of Informatics and Data Mining, Department of Computer and Information Science, Fordham University, New York, NY USA; 2 Program for the Human Environment, The Rockefeller University, New York, NY USA

**Keywords:** Combinatorial fusion analysis (CFA), Decision-making, Visual cognition, Rank-score characteristics (RSC) function, Cognitive diversity

## Abstract

When combining decisions made by two separate visual cognition systems, statistical means such as simple average (*M*_1_) and weighted average (*M*_2_ and *M*_3_), incorporating the confidence level of each of these systems have been used. Although combination using these means can improve each of the individual systems, it is not known when and why this can happen. By extending a visual cognition system to become a scoring system based on each of the statistical means *M*_1_, *M*_2_, and *M*_3_ respectively, the problem of combining visual cognition systems is transformed to the problem of combining multiple scoring systems. In this paper, we examine the combined results in terms of performance and diversity using combinatorial fusion, and study the issue of when and why a combined system can be better than individual systems. A data set from an experiment with twelve trials is analyzed. The findings demonstrated that combination of two visual cognition systems, based on weighted means *M*_2_ or *M*_3_, can improve each of the individual systems only when both of them have relatively good performance and they are diverse.

## Introduction

Many decisions that humans have to make are partially, or even wholly, based on visual input. The split second nature of such decisions may make the process seem simple. However, there are many factors that are considered and combined during this short time frame. On a neurological level, there has been growing interest in understanding the factors that are combined within the visual aspect alone [1, 2], as well as how visual information is joined with information from other senses [3–7]. Combination of multiple visual decisions has also been explored [5, 8, 9].

Prior research into how pairs of people can interactively make decisions based on visual perception has been conducted by several researchers including Bahrami et al. [8], Ernst [5], and Kepecs et al. [9]. In Bahrami’s work, four predictive models are used on experiments of varying degrees of noise, feedback, and communication: coin-flip (CF), behavioral feedback (BF), weighted confidence sharing (WCS), and direct signal sharing (DSS). Bahrami concludes that the WCS model is the only one that can be fit over the empirical data. His findings indicate that the accuracy of the decision-making is aided by communication between the pairs and can greatly improve the overall performance of the pair.

Marc O. Ernst expands on the concept of WCS [5] between pairs by proposing a hypothetical soccer match during which two referees determine whether the ball falls behind a goal line. Similar to Bahrami’s proposal, Ernst’s findings indicate that simply taking the approach of BF or a CF omits information which could lead to an optimal joint decision between the pair. However, while Ernst agrees that the WCS model can lead to a beneficial joint determination, his findings also indicate that there are improvements that can be made to the WCS model to achieve a more optimal joint decision. With Ernst’s scenario, Bahrami’s WCS model can be applied as the distance of the individual’s decision (*d*_*i*_) divided by the spread of the confidence distribution (*σ*), which is *d*_*i*_/*σ*_*i*_. A modified version of WCS (which closely resembles DSS) using sigma-square can produce a more accurate estimate through the joint opinion, which is represented as *d*_*i*_/*σ*_*i*_^2^. In an affirmation of Bahrami’s research, Ernst also notes that joint decision-making comes with a cost when individuals with dissimilar judgments attempt to come to a consensus in such a manner. Bahrami and Ernst set forth very different experimental methods, but their aim is very much the same: to devise an algorithm for optimal decision-making between two people based on visual sensory input.

In the other direction, neural bases for decision-making and combining sensory information within senses have been studied by Gold and Shadlin [10] and Hillis et al. [1]. Koriat [11] indicated that there is no need to combine two heads’ decisions under a normal environment. His suggestion is to simply take the decision of the most confident person.

Combinatorial Fusion Analysis (CFA), an emerging information fusion paradigm, was proposed for analyzing the combination of multiple scoring systems (MSS) (see Hsu et al. [12–14]). CFA has been shown to be useful in several research domains, including sensor feature selection and combination [15, 16], information retrieval, system selection and combination [12, 17], text categorization [18], protein structure prediction [19], image recognition [20], target tracking [21], ChIP-seq peak detection [22], and virtual screening [23]. These studies have shown in its respective domain that combination of MSS performs better than individual systems when the individual scoring systems perform relatively well and they are characteristically different [13, 14].

In a series of previous studies [24–26], a modified version of the soccer goal line decision proposed by Ernst is used as the data collection method. In this experiment, two subjects observe a small target being thrown into a grass field. The subjects are separately asked of their decision on their perceived landing point of the target and their respective confidences in their decisions. More recently, we conducted two sets of experiments with a total of 20 trials on two different days (12 trials and 8 trials) [27, 28]. In each of these trials, a small token was thrown into a grass field and landed at location *A* = (*A*_*x*_, *A*_*y*_). Two subjects *P* and *Q* standing 40 feet away from the landing site would perceive the landing site as at location *P* = (*P*_*x*_, *P*_*y*_) and *Q* = (*Q*_*x*_, *Q*_*y*_) with confidence radius *σ*_*P*_ and *σ*_*Q*_, respectively. In these works, each visual cognition system is treated as a scoring system which assigns a score to each of the partitioned intervals in the common visual space. Then the problem of combining visual cognition systems is transformed to the problem of combining multiple scoring systems. The combination is analyzed using the CFA framework. Results obtained showed that combination by rank as well as by score can improve individual systems.

In this paper, we explore the issue of when and why a combination of two cognitive systems is better than each individual system using the CFA. In particular, we use the concept of “cognitive diversity” and the notion of “performance ratio” to analyze the outcome of the combination. Using the data set from the experiment with twelve trials [27], we demonstrate, as in other domain applications, that combination is positive (better than or equal to the best of the two individual systems) only if the two systems, based on weighted mean using confidence radius, are relatively good (higher performance ratio) and they are diverse (higher cognitive diversity).

Section 2 of this paper discusses two methods of combining visual cognition systems: statistical mean and combinatorial fusion. In Sect. 2.1, three statistical means *M*_1_, *M*_2_, and *M*_3_ are calculated as average or weighted mean using the confidence radius as the weight. Based on these means, scoring systems *p* and *q* are constructed from the two visual cognition systems *P* and *Q*, respectively, in Sect. 2.2. Section 2.3 gives the method to combine these two visual scoring systems using the CFA framework. Section 3 gives the definition of cognitive diversity and the notion of performance ratio. Section 4 consists of examples, in particular the data set of an experiment with twelve trials of pairs of visual cognition systems [27]. Combination of these two visual cognition systems and analysis of the combination for the data set is discussed in more detail in Sect. 4.2 and 4.3. A summary of the results and possible future works is discussed in Sect. 5.

## The CFA framework for combining two visual cognition systems

### Computing various statistical means

When we make a decision based on visual input, we can consider this decision-making as a contemplation of various choices or candidates. Given two perceived locations *P* = (*P*_*x*_, *P*_*y*_) and *Q* = (*Q*_*x*_, *Q*_*y*_) (with confidence radius *σ*_*P*_ and *σ*_*Q*_, respectively) of the actual landing site *A* = (*A*_*x*_, *A*_*y*_), we wish to find a new location L (obtained by the joint decision of *P* and *Q*) so that *L* is better than *P* and *Q* (distance between *L* and *A* is smaller than those between *P* and *A*, and *Q* and *A*). When determining a joint decision, typically an average or a weighted average approach is used to determine a mean. Average mean *M*_1_ = (*M*_1*x*_, *M*_1*y*_) of the two locations *P* = (*P*_*x*_, *P*_*y*_) and *Q* = (*Q*_*x*_, *Q*_*y*_) is calculated as

1 $$ M_{ 1} = \, \left( {P \, + \, Q} \right) \, /{ 2 }, $$

and weighted means are obtained by

2 $$ M_{ 2} = \, \left( {P/\sigma_{P} + \, Q/ \, \sigma_{Q} } \right) \, / \, \left( { 1/\sigma_{P} + { 1}/ \, \sigma_{Q} } \right), $$

and

3 $$ M_{ 3} = \, \left( {P/\sigma_{P}^{ 2} + \, Q/ \, \sigma_{Q}^{ 2} } \right) \, / \, \left( { 1/\sigma_{P}^{ 2} + { 1}/ \, \sigma_{Q}^{ 2} } \right), $$

where *P* and *Q* are the perceived locations of the individual subjects *P* and *Q*, and *σ*_*P*_ and *σ*_*Q*_ are the confidence measurement of the two subjects, respectively.

### Converting each visual cognition system to a scoring system

In the experiments we conducted, each of the two subjects provides an individually determined decision on where they respectively perceived the same target has landed in a field. Each coordinate on the field can be considered as a candidate for the respective participants’ decisions of the perceived landing point. We are able to obtain a weight for each decision and their combination by asking each subject of a radius measurement of confidence around his or her decision. The smaller the radius measure of confidence, the more confident is the participant. We use radius R to calculate the spread (i.e., standard deviation) of the distribution around the perceived landing point, or *σ*. In our research, we use

4 $$ \sigma = \, 0. 5 {\text{R}}. $$

#### Set common visual space

The σ values are used in Formulas (1), (2), and (3) to determine the positions of the means and denoted as *M*_1_, *M*_2_, and *M*_3_ respectively. The distance between *M*_i_ and *A*, *m*_i_ = d(*M*_i_, *A*), where *A* is the actual landing site, is used to evaluate the performance of *M*_i_. With the field used as a two-dimensional coordinate grid, *P*, *Q*, and *A* are represented as *x*- and *y*- coordinates. Three formulas are used to calculate the mean of *P* and *Q*, as *M*_*i*_, where *i* = 1, 2, or 3. *M*_*i*_ falls somewhere in between points *P* and *Q* and is determined as a coordinate.

**Fig. 1:**
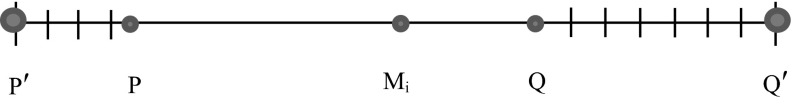
The extension of *PQ* to *P*′*Q*′ based on *M*_*i*_ for *i* = 1, 2, or 3

The longer of either segment *PM*_*i*_ or *M*_*i*_ *Q* is extended 30 % to the left to point *P*′ or to the right to point *Q*′, respectively. The shorter side is extended more to create the widened observation area *P*′*Q*′ so that M_i_ is the midpoint of *P*′ and *Q*′. We refer to the line segment *P*′*Q*′ as the common visual space (Fig. 1).

We partition the length, d(*P*′,*Q*′), of line segment *P*′*Q*′ into 127 intervals with midpoint d_i_ in each interval *i*, *i* = 1, 2, …, 127, and with each interval length d(*P*′,*Q*′)/127. The midpoint of the center interval, in this case, d_64_, contains *M*_*i*_.

#### Treat *P* and *Q* as two scoring systems *p* and *q*

**Fig. 2:**
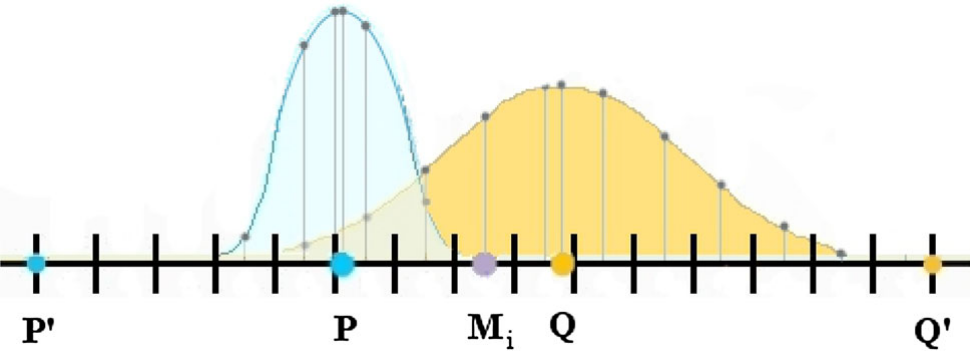
Partition of *P*′*Q*′ into 15 intervals with center *M*_*i*_

**Fig. 3:**
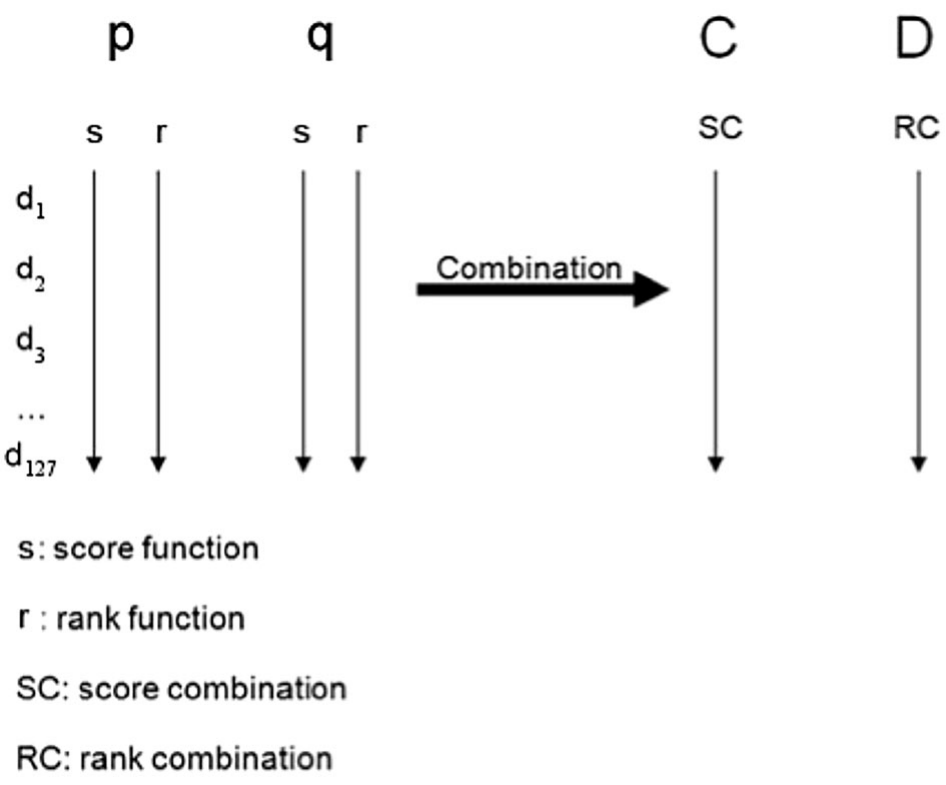
Score and rank function for respective scoring systems *p* and *q* undergo CFA to produce score combination *C* and rank combination *D*

Normal distribution probability curves for each participant are created with the point *P* and *Q* as the mean and using the confidence radii values, *σ*_*P*_^2^ and *σ*_*Q*_^2^ of *P* and *Q* as the variances of *P* and *Q*, respectively (see Fig. 2 in the case of 15 intervals). The following formula is used to determine normal distribution:

5 $$ Y \, = \, \left( { 1 { }/ \, \left( {\sigma \surd \left( { 2\pi } \right)} \right)} \right) * {\text{e}}^{{\left[ { - \, \left( {x \, - \, \mu } \right)** 2} \right]/{ 2}\sigma ** 2}} , $$

where *x* is a normal random variable, *μ* is the mean, and *σ* is the standard deviation. A normal distribution curve spans infinitely to the right and to the left. Therefore, our two scoring systems *p* and *q* create overlapping distributions that span the entire visual plane between *P*′ and *Q*′. Scoring system p and scoring system q, respectively, scores each of the 127 intervals on the common visual space. For normal distribution functions with point *P* and *Q* as the mean and *σ*_*P*_ and *σ*_*Q*_ as the standard deviation respectively, each of the scoring systems p and q assigns interval d_i_ a score between 0 and 1 according to formula (5) (see Fig. 2 in the case of 15 intervals). These are the score functions *s*_*p*_ and *s*_*q*_. The values of the score function s are sorted from highest to lowest to obtain the rank functions *r*_*p*_ and *r*_*q*_, respectively (see Fig. 3). The *d*_*i*_ with the lowest integer as its rank has the highest score.

### Combining scoring systems *p* and *q* using both score and rank combination

Let *D* be a set of candidates with |*D*| = *n*. Let *N* = [1, *n*] be the set of integers from 1 to *n* and R be a set of real numbers. In the context of a CFA framework, a scoring system *A* consists of a score function *s*_*A*_ and a rank function *r*_*A*_ on the set *D* of possible n positions (in this paper, *D* = {*d*_*i*_| *i* = 1, 2, …, 127}).

In the setting of this paper, the score function *s*_*C*_ of the score combination of derived scoring systems p and q in our experiment is

6 $$ s_{C} \left( {d_{i} } \right) = \left( {s_{p} \left( {d_{i} } \right) + s_{q} \left( {d_{i} } \right)} \right) \, / 2 { }. $$

The score function *s*_*D*_ of the rank combination of the two scoring systems *p* and *q* in our experiment is

7 $$ s_{D} \left( {d_{i} } \right) = \left( {r_{p} \left( {d_{i} } \right) + r_{q} \left( {d_{i} } \right)} \right) \, /{ 2}. $$

**Fig. 4:**
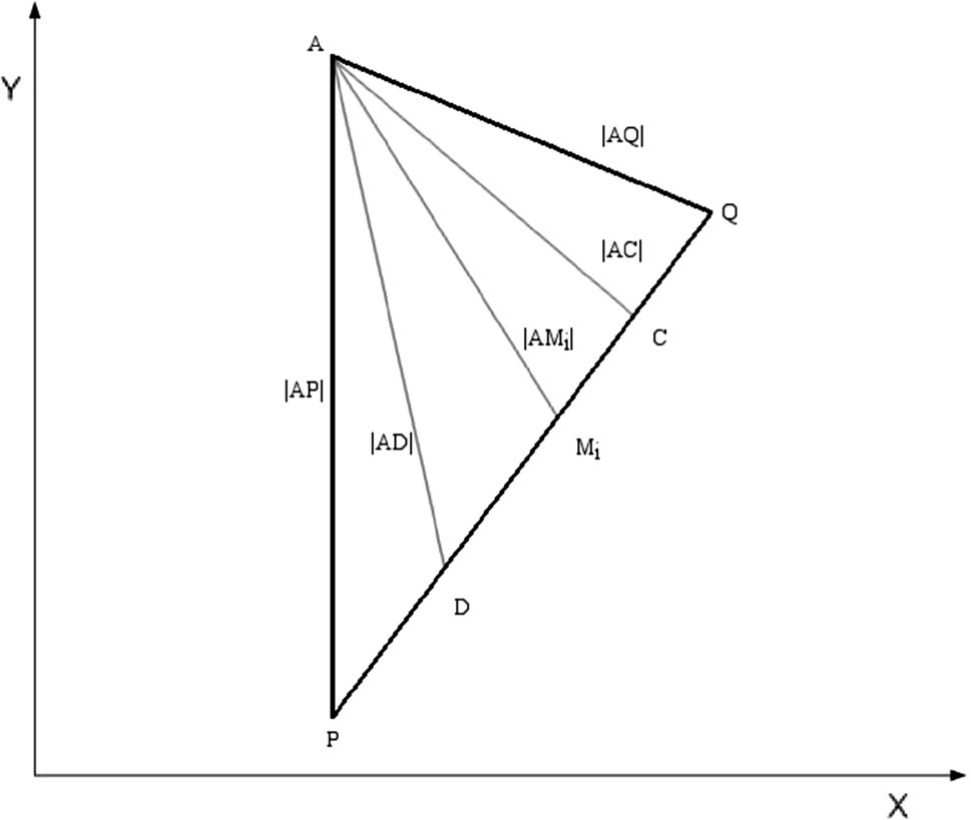
Layout of M_*i*_, *i* = 1, 2, or 3, *C*, and *D* in relation to *P*, *Q*, and their distance to *A*. The distances between the 5 estimated points and *A* are noted on each line [24]

When we sort *s*_*C*_(*d*_*i*_) in descending order, we obtain the rank function of the score combination, called *r*_*C*_(*d*_*i*_). When we sort *s*_*D*_(*d*_*i*_) in ascending order, we obtain the rank function of the rank combination, called *r*_*D*_(*d*_*i*_). The top ranked interval in *r*_*C*_(*d*_*i*_) is called *C*. The top ranked interval in *r*_*D*_(*d*_*i*_) is called *D* (see Fig. 3). These points are considered the optimal score and rank combination, respectively, and are used for evaluation of the combination result. The performance of the points (*P*, *Q*, *M*_*i*_, *C*, and *D*) is determined by each respective point’s distance from target *A*. *A* shorter distance indicates higher performance (Fig. 4).

**Fig. 5:**
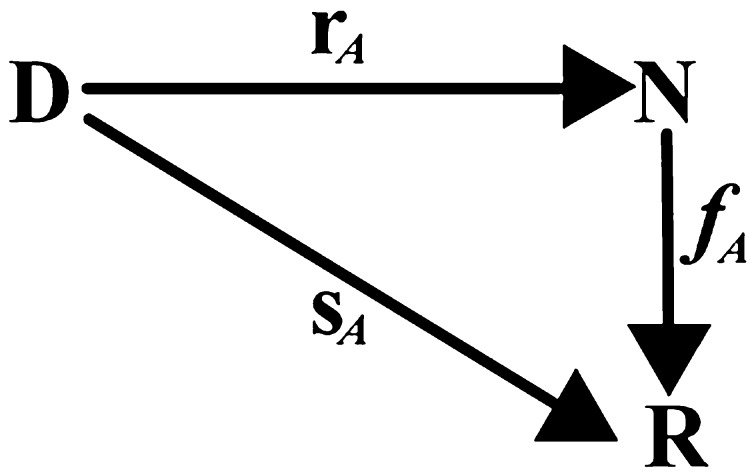
Score function *s*_*A*_, rank function *r*_*A*_, and RSC function *f*_*A*_ of the scoring system *A* [13, 14]

## Cognitive diversity and performance ratio

### Cognitive diversity

Given the score function *s*_*A*_ of the system *A* and its derived rank function *r*_*A*_, rank-score characteristic (RSC) function *f*_A_, which is a composite function of *s*_*A*_ and the inverse of *r*_*A*_, defined by Hsu et al. [13, 14] is a function from *N* to *R* and can be computed mathematically as (see Fig. 5).

8 $$ f_{A} \left( i \right) = (s_{A} r_{A}^{ - 1} )\left( i \right) \, = \, s_{A} (r_{A}^{ - 1} \left( i \right)). $$

The cognitive diversity between two scoring systems *p* and *q*, d(*p*,*q*) is calculated using RSC functions *f*_*p*_ and *f*_*q*_ (also see [23]) as

9 $$ {\text{d}}\left( {p,q} \right) = {\text{d}}(f_{p} ,f_{q} ) = \left( {\sum\limits_{i = 1}^{127} {( f_{p} \left( i \right) - f_{q} (i))^{2} } /127} \right)^{1/2} . $$

### Performance ratio

The performances of each *P* and *Q* for all trials are used in calculating the performance ratio. Performance of *P* (or *Q*) is determined by the distance between *P* (or *Q*) and *A*, d(*P*, *A*) [or d(*Q*, *A*)], respectively. Shorter distance indicates high performances. Each distance is inverted and then multiplied by the maximum distance *md* = max{d(*P*_*i*_, *A*_*i*_), d(*Q*_*i*_, *A*_*i*_) | *i* = 1, 2,…, 12} for all trials. Let ${\text{MAX = max}}\left\{ {\frac{md}{{{\text{d}}(P_{i} ,   A_{i} )}}, \frac{md}{{{\text{d}}(Q_{i} , A_{i} )}}\left| {i = 1,2, \ldots ,12} \right.} \right\}$. Then this set of numbers is each divided by MAX. In this way, the performance for each of the 12 *P* and *Q* is in the set (0, 1]. The smaller performance over the higher performance for *P* and *Q* is the performance ratio after it is normalized again among the twelve ratios to be in (0, 1].

## Example

### Data set

We use the data set from an experiment of twelve trials conducted by the authors in [27]. Each trial consists of two volunteers *P* and *Q* with confidence radius *σ*_*P*_ and *σ*_*Q*_. Each gives a visual cognitive estimate of the actual token landing site *A* as *P* and *Q* respectively.

Table 1 lists coordinates of *P* (*P*_*x*_, *P*_*y*_), *Q* (*Q*_*x*_, *Q*_*y*_), and *A* (*A*_*x*_, *A*_*y*_) as well as the confidence radius *σ*_*P*_ and *σ*_*Q*_ of *P* and *Q* respectively.

**Table 1:** Coordinates of *P*, *Q*, and *A* and confidence radius (*σ*) of *P* and *Q* for the 12 trials [27]

Trial	(*P*_*x*_, *P*_*y*_)	*σ*_*P*_	(*Q*_*x*_, *Q*_*y*_)	*σ*_*Q*_	(*A*_*x*_ *, A*_*y*_)
1	(11.5, 134.5)	11.5	(78.5, 105)	16	(94, 124)
2	(23.5, 56)	7	(112, 96.75)	21.5	(28.5, 43)
3	(105, 134.25)	21	(78.5, 87.75)	22	(39.5, 119)
4	(229.25, 151.5)	14	(256, 162.5)	15.5	(216.25, 149.75)
5	(125.5, 13.5)	0.5	(112.75, 57.25)	3	(113.75, 46)
6	(184.5, 108.25)	21.5	(164.5, 249.75)	12	(173.25, 212.5)
7	(22, 190.5)	7	(17, 227.75)	6	(14.75, 195)
8	(98.75, 57)	12.5	(71.25, 25.5)	12	(16.5, 1)
9	(205.5, 15)	17	(204, 21.5)	6.5	(203, 26)
10	(100.5, 4.5)	19.5	(172, 25.25)	6	(127, 9.5)
11	(236.25, 43)	4	(234, 72.75)	4.5	(229, 51.5)
12	(98.5, −75.5)	10	(99, 30)	12	(96, 4)

### Combination results and analysis

The decision of Participant *p*, marked as *P*, and the decision of Participant *q*, marked as *Q*, are used to obtain line segment *PQ*. The radii of confidence are used to calculate the two *σ* values to locate the coordinates of points *M*_1_, *M*_2_, and *M*_3_ along the extended *P*′*Q*′. To combine and compare the two visual decision systems of *p* and *q*, a common plane must be implemented to be evaluated by the different systems. The 127 intervals along the *P*′*Q*′ line serve as the common visual space to be scored.

When *P*′*Q*′ has been partitioned into the 127 intervals mapped according to *M*_*i*_, the intervals are scored according to the normal distribution curves of *P* and *Q* using the standard deviation *σ*_*P*_ and *σ*_*Q*_, respectively. Both systems assume the set of common interval midpoints *d*_1_, *d*_2_, *d*_3_,…,*d*_127_. Each scoring system, *p* and *q*, consists of a score function. We define score functions *s*_*P*_(*d*_*i*_) and *s*_*Q*_(*d*_*i*_) that map each interval, *d*_*i*_, to a score in systems *p* and *q*, respectively. The rank function of each of the systems *p* and *q* maps each element *d*_*i*_ to a positive integer in *N*, where *N* = {x | 1 ≤ x ≤ 127}. We obtained the rank functions *r*_*P*_(*d*_*i*_) and *r*_*Q*_(*d*_*i*_) by sorting *s*_*P*_(*d*_*i*_) and *s*_*Q*_(*d*_*i*_) in descending order and assigning a rank value from 1 to 127 to each interval. *C* and *D* based on *M*_*i*_, for *i* = 1, 2, and 3, are calculated, and the distances to target *A* are computed. The point with the shorter distance from the target is considered the point with the better performance.

Table 2 lists the performance of (*P*, *Q*), confidence radius of *P*, *Q* and performance of *C* and *D* based on *M*_*i*_, *i* = 1, 2, and 3. Table 3 lists performance for *M*_*i*_, *i* = 1, 2, and 3 in the twelve trials. Table 4 gives comparisons of the performance of *C* or *D* to that of *P* and *Q*, and to *M*_*i*_. We note that Koriat’s criterion, taking the decision of the most confident system, gives a correct prediction of 7 out of the 12 trials (Trials 1, 2, 4, 6, 8, 9, and 11). The score combination *C* or rank combination *D* obtained by CFA improves *P* and *Q* in 8, 7, and 6 out of the 12 trials when the common visual space mean is *M*_1_, *M*_2_, and *M*_3_ respectively. It is interesting to note that *C* or *D* improves *P* and *Q* in more trials based on *M*_1_ than those based on *M*_2_ or *M*_3_ because *M*_1_ does not take into consideration the confidence radius as weighted means (Table 4(a)). The same reason can be given to Table 4(b) where *C* or *D* can improve *M*_1_ in more trials than M_2_ or M_3_. In addition, in the 4 trials (Trials 3, 5, 10, and 12) that Koriat’s criterion fails to apply, they can all be improved using the CFA framework.

**Table 2:** Performance of combination: (a) Performance of *P*, *Q*, (b) Confidence radius of *P*, *Q*, (c) Performance of *C* and *D* based on *M*_1_, *M*_2_, and *M*_3_, respectively

Trial	(a) Per. (*P*,*Q*)	(b) Confidence Radius (*σ*_*P*_, *σ*_*Q*_)	(C)(1) Per. of C, D; based on *M*_1_	(C)(2) Per. of C, D; based on *M*_2_	(C)(3) Per. of C, D; based on *M*_3_
1	(**20.41**, 24.52)	(**11.5**, 16)	(**20.24**, **20.24**)	(20.63, **20.07**)	(**20.14**, **20.14**)
2	(**13.93**, 99.3)	(**7**, 21.5)	(13.96, 13.96)	(**13.91**, **13.91**)	(**13.91**, **13.91**)
3	(67.25**, 49.98**)	(**21**, 22)	(66.71, **49.94**)	(66.72, 67.13)	(66.70, 67.15)
4	(**13.12,** 41.74)	(**14**, 15.5)	(14.47, 13.23)	(14.40, **13.11**)	(14.48, 13.19)
5	(34.56**, 11.29**)	(**0.5**, 3)	(34.38, **11.12**)	(**10.95**, **10.95**)	(34.51, **10.94**)
6	(104.86, **38.26**)	(21.5**, 12**)	(**37.70**, **37.70**)	(**37.63**, **37.63**)	(**37.95**, **37.95**)
7	(**8.53,** 32.83)	(7, **6**)	(32.68, **8.44**)	(32.88, 32.44)	(32.68, 32.68)
8	(99.5, **59.98**)	(12.5, **12**)	(60.13, 60.13)	(**59.79**, **59.79**)	(**59.90**, **59.90**)
9	(11.28, **4.61**)	(17, **6.5**)	(4.86, 4.64)	(4.86, 4.65)	(4.95, **4.56**)
10	(**26.97**, 47.68)	(19.5, **6**)	(47.38**, 26.68**)	(47.73, 46.48)	(47.24, 47.24)
11	(**11.17**, 21.83)	(**4**, 4.5)	(**11.08**, **11.08**)	(**11.08**, **11.08**)	(11.22, **10.92**)
12	(79.54**, 26.17**)	(**10**, 12)	(79.12, **25.76**)	(79.80, 78.53)	(78.86, 78.86)

Bold numbers indicate *C* and/or *D* perform better than *P* and *Q* in (C)(1), (C)(2) and (C)(3). Bold numbers indicate better performance of the two systems in (a) and higher confidence in (b)

**Table 3:** Performance of *M*_1_, *M*_2_, *M*_3_ in 12 trials

	Trial											
	1	2	3	4	5	6	7	8	9	10	11	12
*M*_1_	**4.37**	51.52	52.86	27.35	11.91	**33.52**	14.90	79.45	7.95	**10.70**	**8.84**	26.89
*M*_2_	**4.13**	28.45	53.08	26.62	33.09	**13.53**	16.21	79.05	6.45	30.20	**8.28**	31.66
*M*_3_	**6.28**	17.26	53.32	25.90	34.51	**5.41**	17.53	78.64	5.46	41.25	**7.78**	36.36

Each bold number indicates the performance of *M*_*i*_ in the Trial is better than *P* and *Q*. *M*_3_ is best among *M*_*i*_’s in Trials 2, 4, 6, 8, 9, and 11

**Table 4:** Comparisons of performance of C or D to that (a) of *P* and *Q*, (b) of *M*_i_, and (c) of *P*, *Q*, and *M*_i_ (set of 36 cases in Table 2)

	(a) *C* or *D* ≥ *P* and *Q*	(b) *C* or *D* ≥ *M*_*i*_	(c) *C* or *D* ≥ *P*, *Q*,& *M*_*i*_
*M*_1_	1, 3, 5, 6, 7, 10, 11, 12 (8/12)	2, 3, 4, 5, 7, 8, 9, 12 (8/12)	3, 5, 7, 12 (4/12)
*M*_2_	1, 2, 4, 5, 6, 8, 11 (7/12)	2, 4, 5, 8, 9 (5/12)	2, 4, 5, 8 (4/12)
*M*_3_	1, 2, 5, 6, 8, 9 (6/12)	2, 4, 5, 8, 9 (5/12)	2, 5 (2/12)
Total	**21/36**	**18/36**	**10/36**

**Fig. 6:**
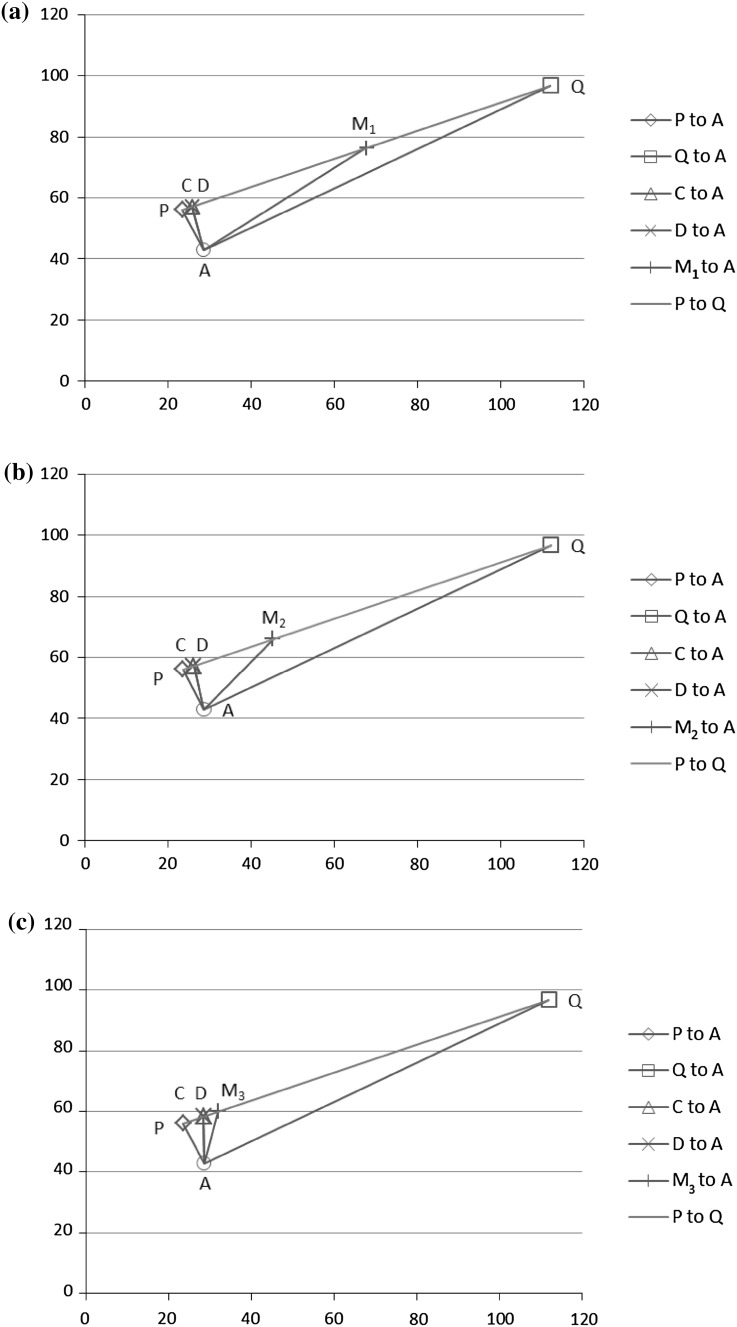
Performance of *P*, *C*, *D*, and *Q* based on *M*_1_ (**a**), *M*_2_ (**b**), and *M*_3_ (**c**) respectively for Trial 2, **a** Performance of *P*, *Q*, *C*, and *D* based on *M*_1_ in Trial 2, **b** performance of *P*, *Q*, *C*, and *D* based on *M*_2_ in Trial 2, **c** performance of *P*, *Q*, *C*, and *D* based on *M*_3_ in Trial 2

**Fig. 7:**
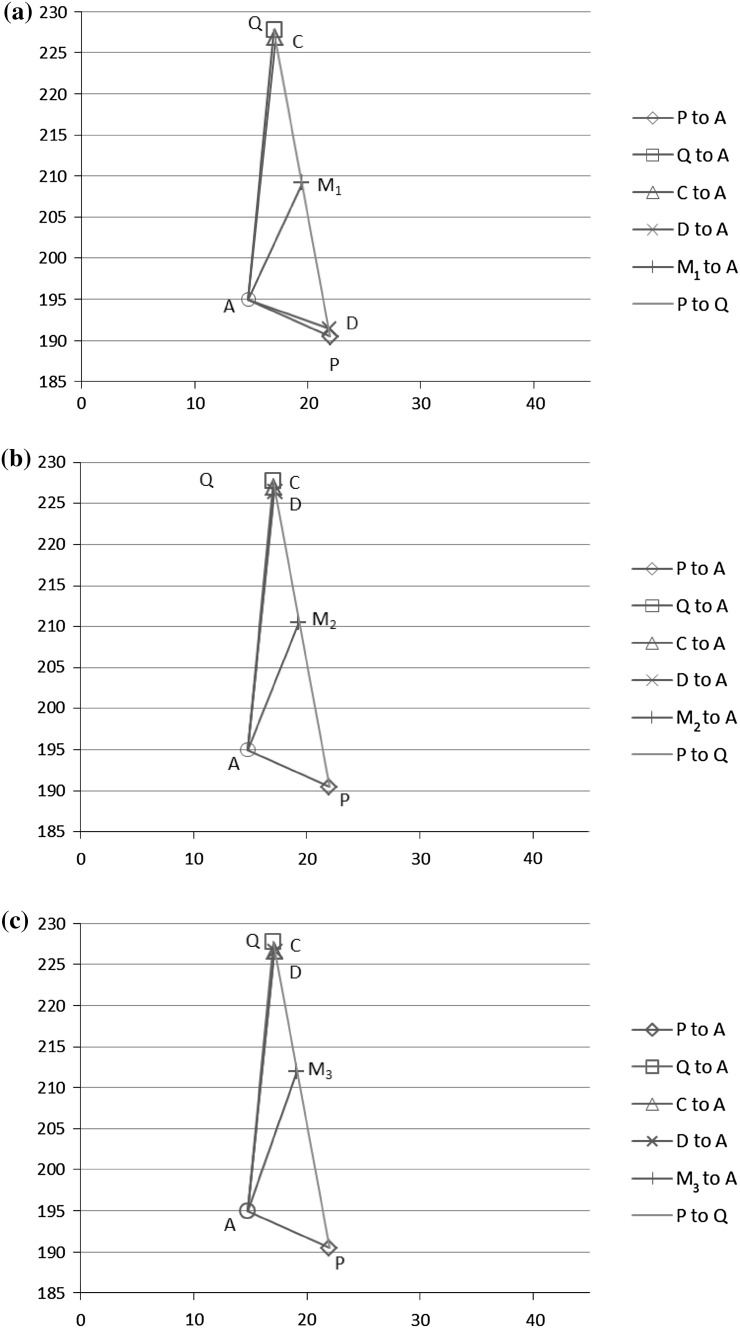
Performance of *P*, *C*, *D*, and *Q* based on *M*_1_ (**a**), *M*_2_ (**b**), and *M*_3_ (**c**) respectively for Trial 7, **a** Performance of *P*, *Q*, *C*, and *D* based on *M*_1_ in Trial 7, **b** performance of *P*, *Q*, *C*, and *D* based on *M*_2_ in Trial 7, **c** performance of *P*, *C*, *D*, and *Q* based on *M*_3_ in Trial 7

Figures 6 and 7 illustrate the performances of *P*, *C*, *D*, *M*_*i*_ and *Q* for *i* = 1, 2, and 3 in Trials 2 and 7 respectively. In Trial 2, *P* performs quite good and has a higher confidence radius than *Q*. When given weighted means *M*_2_ and *M*_3_, combinatorial fusion *C* or *D* performs better than *P* and *Q*. However, in Trial 7, *P* performs better but has a lower confidence radius than *Q*. In this case, *C* or *D* does not improve *P* and *Q* based on *M*_2_ or *M*_3_ when more weight is given to *Q*. Therefore, we observe that giving more weight to the better performer with a higher confidence leads to a combination which improves *P* and *Q*. We call such a case a positive case. In the following Sect. 4.3, we investigate in general when combination (either rank or score combination) can improve *P* and *Q*.

### Positive cases versus Negative cases

We plot the result of a score or rank combination of *P* and *Q*, distinguishing positive cases as “□” or “◊” and negative cases as “×” or “+” on the two-dimensional coordinate plane with the y-axis as the cognitive diversity d(*P*, *Q*) and the x-axis as the performance ratio *P*_l_/*P*_h_ (lower performance over higher performance) for all the trials for each *M*_*i*_, *i* = 1, 2, or 3. Each trial within each graph is noted as positive when rank or score combination performs better than both *P* and *Q*, and negative when it does not. The average for all positive cases and the average for all negative cases is also marked for each graph as “■” and “**X**” respectively.

**Fig. 8:**
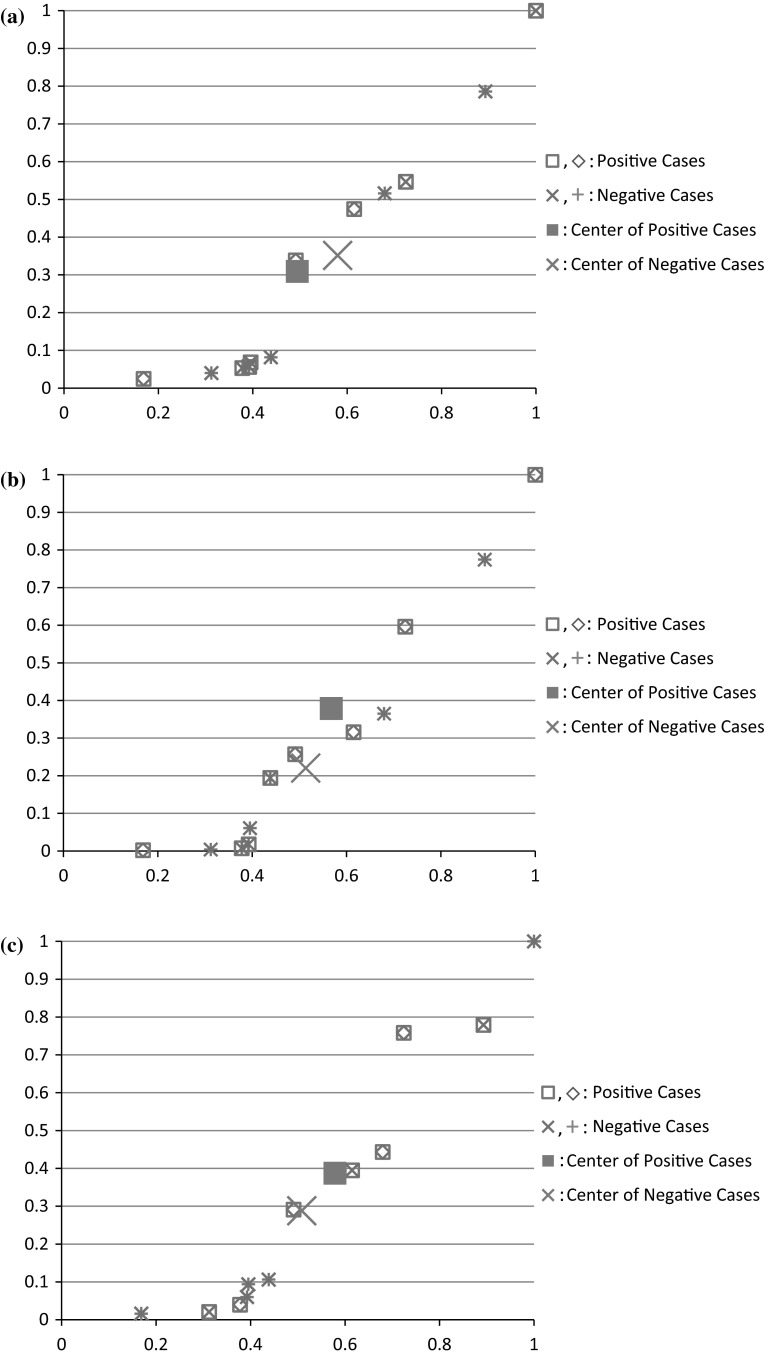
Positive versus negative cases resulting from the 24 score and rank combinations in terms of cognitive diversity d(*P*, *Q*) (y-axis) and performance ratio *P*_l_/*P*_h_ (x-axis) based on *M*_1_ (**a**), *M*_2_ (**b**), and *M*_3_ (**c**) respectively, **a** Positive versus negative cases based on *M*_1,_ **b** positive versus negative cases based on *M*_2,_ **c** positive versus negative cases based on *M*_3_

Cognitive diversity between *P* and *Q*, d(*P*, *Q*), is the diversity between two RSC functions *f*_*p*_ and *f*_*q*_, d(*f*_*p*_, *f*_*q*_), and is calculated using formula (9). Cognitive diversity values are normalized to (0, 1] in each case based on *M*_*i*_, *i* = 1, 2, and 3 (see Table 5). Figure 8 depicts the positive versus negative cases based on each M_*i*_, *i* = 1, 2, and 3 (Fig. 8a–c respectively) in terms of cognitive diversity (y-axis) and performance ratio (x-axis).

**Table 5:** Cognitive diversity

Trial	d(*p*, *q*) in *M*_1_	d(*p*, *q*) in *M*_2_	d(*p*, *q*) in *M*_3_
1	0.338434959	0.194412684	0.291268428
2	0.785773308	0.596314746	0.758254198
3	0.056297571	0.003988946	0.059975847
4	0.081718215	0.007480963	0.106193355
5	0.546649181	0.257373029	0.394914152
6	0.474573259	0.315880355	0.443491266
7	0.053300385	0.017943343	0.016196129
8	0.040005652	0.002402607	0.039874004
9	0.516003209	0.36502678	0.779226911
10	1	0.774343099	1
11	0.024840875	1	0.02068517
12	0.068319595	0.060741956	0.093857643

## Summary and future work

In our previous work [27, 28], it has been demonstrated that combination of two visual cognition system using the CFA framework can improve each of the individual systems. In this paper, we analyze outcomes of these combinations according to positive cases or negative cases using the notions of cognitive diversity and performance ratio on the data set of an experiment with 12 trials [27]. It is demonstrated that in the majority of the 72 cases of rank combinations and score combinations (12 × 2 × 3 = 72) (see Fig. 8a–c), combination of two visual systems, based on weighted means *M*_2_ or *M*_3_, can outperform each of the individual systems only if they each perform relatively well (with higher performance ratio) and they are diverse (with high cognitive diversity).

In an earlier work by Hsu and Taksa [12], it was shown that under certain conditions, rank combination can be better than score combination. In the current study, each of the six trials (Trials 1, 2, 5, 6, 9, and 10) has higher diversity than the remaining six trials. Similar to the results in [12], the six trials do have better rank combination (*D*) than score combination (*C*). It is also interesting to note that improvement in the other six trials was carried out by rank combination only (Trial 3, 4, 7, 8, 11, and 12). In other cases, whenever score combination (*C*) improves *P* and *Q*, rank combination (*D*) can also improve. All these indicate that the CFA framework, which uses score and rank combination, is robust in analyzing combination and decision problems for visual cognition systems.

In the combination of decisions or visual cognition systems, as well as the integration of signals from different sensors, statistical means or weighted means such as *M*_1_, *M*_2_, or *M*_3_ are often used [1, 3, 4, 5, 8]. It has been observed in these previous studies that *M*_3_, using 1/*σ*_*P*_^2^ (or 1/*σ*_*Q*_^2^) as the weight assigned to system *P* (or *Q*), provides better combination results. In our current study, when comparing *M*_1_, *M*_2_, and *M*_3_ in each of the 12 trials, it is shown that *M*_3_ is better than *M*_1_ and *M*_2_ in 6 of the 12 trials, while *M*_1_ and *M*_2_ are the best in 5 and 1 of the 12 trials respectively, independent of the performance of *P* and *Q*. So our current study supports that observation. However, when comparing improvements of *M*_*i*_ over *P* and *Q*, it was shown in our study that the statistical means *M*_1_, *M*_2_, and *M*_3_ can improve *P* and *Q* in 4, 3, and 3 trials, respectively (see Table 3). On the other hand, the CFA framework (C or D) based on M_1_, M_2_, or M_3_ can improve *P* and *Q* in 8, 7, or 6 trials. All these indicate that the CFA framework is a viable analytic method in combining visual cognition systems and can be generalized to analyze data in bioinformatics and neuroscience.

In summary, our CFA framework provides two criteria: performance ratio and cognitive diversity to guide us to combine two visual cognition systems with confidence radii. In the case of unsupervised learning or when the performance cannot be evaluated (e.g., the location of *A* is not known), cognitive diversity itself can be used to direct us when to combine (when the cognitive diversity is big enough) or how to combine (use rank combination or score combination) (see [12, 14, 21, 22, and 23]).

Our future work includes the following:

1. Apply CFA framework to the combination of more than two visual systems;
2. Study the effect of the number of partition intervals in the common visual space defined by *P*′*Q*′;
3. Use other diversity measurements such as Pearson’s correlation (between two score functions *s*_*A*_ and *s*_*B*_) and Kendall’s tau (see [29]) or Spearman’s rho (between two rank functions *r*_*A*_ and *r*_*B*_); and
4. Apply CFA framework to combination of multiple sensing systems or combination of multi-modal physiological systems.
